# Depression Among Hemodialysis Patients: Related Factors and the Impact of Insomnia and Fatigue

**DOI:** 10.7759/cureus.25254

**Published:** 2022-05-23

**Authors:** Jona Sakiqi, Georgios Vasilopoulos, Ioannis Koutelekos, Maria Polikandrioti, Evangelos Dousis, Niki Pavlatou, Antonia Kalogianni, Stavros Tsirigotis, Georgia Gerogianni

**Affiliations:** 1 Department of Nursing, Postgraduate Program "Applied Clinical Nursing", University of West Attica, Athens, GRC; 2 Department of Nursing, Postgraduate Program "Applied Clinical Nursing", University of West Attica, Greece, Athens, GRC

**Keywords:** morbidity, fatigue, insomnia, depression, hemodialysis

## Abstract

Introduction: Depression is a commonly experienced psychological disorder among patients undergoing hemodialysis and has a strong association with insomnia and fatigue. The purpose of the present study was to explore the factors related to depression in dialysis patients and the impact of insomnia and fatigue on depression.

Methods: In this cross-sectional study, one hundred (n=100) patients on hemodialysis from one hemodialysis center in Greece participated. Data collection was carried out by interviews using the Zung Self-rating Depression Scale (SDS) for the evaluation of depression; the Athens Insomnia Scale (AIS) for the evaluation of insomnia; the Modified Fatigue Impact Scale (MFIS) for the evaluation of fatigue; and a questionnaire which was created by the researchers. The Kruskal-Wallis, the Mann-Whitney tests, and the Spearman's rho criterion were used to evaluate the relationship between depression score and patient characteristics. Multiple linear regression was performed to assess the effect of characteristics on patients' depression.

Results: Statistically significantly high levels of depression were found in patients over the age of 70 years old (p=0.001), in divorced/widowed patients (p = 0.001), in pensioners (p = 0.002), in patients who had other diseases (p = 0.001), in those who felt tired after hemodialysis or at night and those who felt constantly tired (p = 0.001, p = 0.016, and p = 0.001, respectively), in those who had physical and mental fatigue (p = 0.001 and p = 0.001, respectively), in those who had changes in body image (p = 0.009), itching, stiffness, and nausea/vomiting (p = 0.001, p = 0.001, and p = 0.003, respectively), in patients who had restrictions in clothes they could wear (p = 0.001), in patients who had insomnia (p = 0.001), and in patients who did not wish to receive written information about the management of their disease (p = 0.025). A statistically significant positive linear association was detected between physical/mental fatigue and depression levels (rho = 0.824 and rho = 0.746, respectively) and between the score of insomnia and depression levels (rho = 0.707). An increase in fatigue (either physical or mental) and an increase in insomnia score also indicated an increase in depression levels.

Conclusions: The findings of this study showed that depression had a strong association with a variety of demographic factors. Additionally, an increase in fatigue score and an increase in insomnia score indicated an increase in depression levels. Therefore, depression, insomnia, and fatigue should be early assessed and effectively treated by renal professionals in order to improve the quality of life in hemodialysis patients and reduce their morbidity and mortality.

## Introduction

Depression is a mental disturbance that includes sadness, loss of interest or pleasure, feelings of guilt, interrupted sleep, decreased desire for food, feelings of tiredness, and low concentration [1]. Depression has an increased prevalence in patients with end-stage renal disease (ESRD), ranging from up to 20% to 40% [2]. Depressive disorders in the hemodialysis population are caused by co-morbid diseases, frequent hospital admissions, cognitive impairment, chronic pain, uremic toxins, unemployment, transplant failure, and imminent death [3]. Additionally, functional and nutritional restrictions in daily life, side effects of medication [4], lack of family support, and dependency on health professionals frequently lead to depressive disturbances [5].

Moreover, depression is strongly associated with insomnia and fatigue in hemodialysis patients [6]. More specifically, patients with insomnia have difficulty initiating and maintaining regular sleep while they have tiredness, lack of energy during the day, and increased fatigue [6]. Increased fatigue leads to low levels of physical activity, general muscle weakness, and lack of concentration, which negatively affect patients’ ability to work, take care of their families, and achieve personal goals, leading to emotional problems and depressive disorders [7]. However, early diagnosis of depression in patients undergoing dialysis is difficult since symptoms of depression are usually overlapped with symptoms of other physical conditions, such as uremia, decreased appetite, fatigue, loss of libido, and low sleep quality [4].

The aim of this study was to investigate the factors related to depression in patients undergoing hemodialysis and the impact of insomnia and fatigue on depression.

## Materials and methods

Study sample

The study sample is a convenience sample and consists of 100 dialysis patients in a hemodialysis center in Athens, Greece. Inclusion criteria were: age over 18 years old and less than 85 years old, on hemodialysis for at least three months, and capability to speak, read, and write in Greek. Exclusion criteria were inadequate language skills, age over 85 years old, cognitive decline, and drug or alcohol abuse. Patients who met the inclusion criteria were informed during their treatment about the aim and procedure of the study and the anonymity of the data. Before collecting data, we obtained approval from the Ethics Committee of General Hospital of Athens "Evangelismos - Athens Ophthalmology Clinic - Polyclinic" (Νumber of approval: 587/17-12-2020). The study was carried out from November 2020 to January 2021.

Data collection was carried out by interviews using the Zung SDS for the evaluation of depression, the Athens Insomnia Scale (AIS) for the evaluation of insomnia, the Modified Fatigue Impact Scale (MFIS) for the evaluation of fatigue, and a questionnaire which was created by the researchers and included (a) socio-demographic characteristics: gender, age, marital status, number of children, educational level, occupation; (b) clinical characteristics: whether they suffered from another disease, the degree of information about their health problem, values of urea, creatinine, phosphorus, the level of stress they experienced due to their disease if they wished to receive written information about the management of their disease, fatigue after hemodialysis and its duration, pain after hemodialysis, changes in body image, muscle cramps, stiffness, nausea, vomiting; (c) information about patients' concerns: changes in social and personal life, changes in the volume of fluids and the amount of food they used to take, fear of the possibility of the hemodialysis machine shutting down, difficulties in movement, and in the clothes they could wear. The study was conducted according to the Declaration of Helsinki (1989).

Zung self-rating depression scale

The Zung Self-Rating Depression Scale (SDS) was used to assess depression in patients undergoing hemodialysis. The SDS scale includes 20 questions evaluating the feelings of respondents during the previous week. Participants are able to answer each question on a four-point Likert-type scale. The final score ranges from 20 to 80. Higher scores point out higher scores of depression [8].

Athens insomnia scale

The AIS is a self-assessment psychometric scale assessing difficulty in sleeping. It includes eight questions, the first five of which are related to the onset of sleep, the night's awakenings, the final awakening, the total duration of sleep, and the quality of sleep. The other three questions are associated with wellness, functionality, and fatigue during the day. The total score of insomnia ranges from 0 to 24. Higher scores point out higher scores of insomnia. AIS has high consistency, reliability, and validity in Greek people and is a significant tool in clinical practice [9].

Modified fatigue impact scale

The MFIS is a self-assessment questionnaire and includes 21 questions. Respondents indicate the fatigue they have felt during the last four weeks on a 5-point Likert scale. Ten of the 21 items evaluated the mental fatigue of participants, and the other 11 evaluated physical fatigue. The scores of mental and physical fatigue are summed up separately and range from 10 to 50 for mental fatigue and from 11 to 55 for physical fatigue. High scores point out high levels of mental and physical fatigue, respectively. The MFIS has high reliability and validity among Greek people [10].

Statistical analysis

Nominal data are presented with absolute and relative (%) frequencies, while the continuous data are presented with mean, standard deviation, median, and interquartile range (IQR). The normality of the data was checked with the Kolmogorov-Smirnov criterion and graphically with histograms and Q-Q plots. The Kruskal-Wallis test, the Mann-Whitney test, and Spearman's rho criterion were used to estimate the relationship between depression levels and patient characteristics, insomnia, and fatigue. Multiple linear regression was carried out to evaluate the effect of characteristics, insomnia, and fatigue on patients' depression. The results are presented with β regression coefficients and 95% confidence intervals (CI). The observed significance level of 5% was considered statistically significant. All statistical analyses were performed with version 25 of the SPSS program (SPSS, Inc., Chicago, Il, USA).

## Results

Sample characteristics

A total of 100 patients took part in the research study. Men accounted for 75% of the sample, while 73% were over 60 years old. Additionally, 65% were married, 42% had a primary school education, and 65% were pensioners. The majority of participants had two or more children (63%) (Table 1).

**Table 1:** Demographic characteristics of participants (n=100)

	Ν (%)
Gender	
Male	75 (75.0%)
Female	25 (25.0%)
Age (years)	
30–40	2 (2.0%)
41–50	9 (9.0%)
51–60	16 (16.0%)
61–70	30 (30.0%)
>70	43 (43.0%)
Status	
Single	14 (14.0%)
Married	65 (65.0%)
Divorced	3 (3.0%)
Widowed	17 (17.0%)
Living together	1 (1.0%)
Education level	
Primary school	42 (42.0%)
High school	46 (46.0%)
University	10 (10.0%)
MSc-PhD	2 (2.0%)
Job	
Unemployed	4 (4.0%)
Private employee	3 (3.0%)
Freelancer	16 (16.0%)
Household	9 (9.0%)
Pensioner	65 (65.0%)
Other	3 (3.0%)
Number of children	
0	14 (14.0%)
1	23 (23.0%)
2	45 (45.0%)
>2	18 (18.0%)

Association of depression scale with patients' characteristics

A statistically significant association was observed between patients' depression levels and age (p = 0.001), marital condition (p = 0.001), job (p = 0.002), comorbid diseases of patients (p = 0.001), patients' beliefs that regular updating was helpful in reducing stress (p = 0.016), patients’ desire to receive written information about the management of their illness (p = 0.025), whether they felt tired after dialysis, when they felt tired and for how long (p = 0.001, p = 0.016, and p = 0.001, respectively), whether they had changes in body appearance (p = 0.009), whether they had itching, stiffness and nausea/vomiting (p = 0.001, p = 0.001, and p = 0.003, respectively), whether they had restrictions in clothing (p = 0.001), physical and mental fatigue (p = 0.001 and p = 0.001, respectively) and insomnia (p = 0.001).

More specifically, patients over the age of 70 had statistically significantly higher levels of depression (median 48) than younger patients (median 45 and 37). Divorced/widowed patients had statistically significantly higher levels of depression (median 52) than married (median 44) and single (median 39). Pensioners had higher levels of depression (median 47) than employees (median 37) and unemployed patients (median 41). Patients who had another disease had higher levels of depression (median 51) than those who did not have another disease (median 41). Patients who had the opinion that regular updating did not help decrease stress had statistically significantly high scores of depression (median 53).

Additionally, statistically significantly high scores of depression were found in patients who did not wish to receive written information about the management of their disease (median 51), those who felt tired after hemodialysis (median 54), and those who had increased tiredness at night (median 48), and those who felt constantly tired (median 57), those who felt that they had a change in body appearance (median 48), those who experienced frequent itching (median 53), stiffness (median 56), and sometimes nausea (median 50), and those who had restrictions in clothing (median 48).

Finally, a statistically significant positive linear association was detected between physical/mental fatigue and depression levels (rho = 0.824 and rho = 0.746, respectively) as well as between the score of insomnia and depression (rho = 0.707). An increase in fatigue score (either physical or mental), as well as an increase in insomnia score, indicated an increase in depression levels (Table 2).

**Table 2:** Association of depression scale with patients' characteristics

	Mean (SD)	Median (IQR)	p-value
Gender			0.098
Male	44.5(9.3)	44(37–53)	
Female	47.7(8.9)	48(43–53)	
Age (years)			0.001
≤60	38.8(8.2)	37(33–44)	
61–70	45.5(8.5)	45(39–51)	
>70	49.3(8.2)	48(44–56)	
Status			0.001
Married/living together	43.7(8.5)	44(37–50)	
Single	42.9(10.2)	39(36–52)	
Divorced/widowed	52.5(7.7)	52(48–58)	
Education level			0.172
Primary School	47.1(9.3)	47(40–55)	
High School	44.6(8.9)	45(38–51)	
University/MSc-PhD	41.8(10.0)	40(34–48)	
Job			0.002
Unemployed/household	42.9(6.8)	41(39–48)	
Employee	39.5(9.1)	37(33–46)	
Pensioner	47.5(9.2)	47(40–56)	
Number of children			0.499
0	42.0(9.9)	39(36–46)	
1	46.3(10.4)	45(37–55)	
2	45.6(8.4)	45(39–51)	
>2	46.0(9.7)	46(38–56)	
Informed about their health problem			0.062
Very	41.0(9.6)	38(36–46)	
Enough	44.9(8.5)	44(38–52)	
A little/not at all	49.0(10.6)	53(45–56)	
Other diseases			0.001
Yes	49.7(9.2)	51(42–57)	
No	41.9(7.8)	41(36–47)	
Are you nervous about the course of the disease?			0.777
Very	45.6(9.4)	45(38–52)	
Enough	44.3(8.2)	44(39–50)	
A little/not at all	46.2(10.4)	47(36–55)	
Do you believe that regular updating helps reduce stress?			0.016
Very	44.4(9.6)	42(37–53)	
Enough	43.0(7.9)	44(37–47)	
A little/not at all	50.0(9.3)	53(44–56)	
Do you wish to receive written information regarding the management of the disease?			0.025
Yes/sometimes	44.1(8.9)	44(37–51)	
No	49.3(9.4)	51(43–56)	
How often do you weigh yourself at home?			0.392
Daily	43.6(8.5)	45(39–49)	
Every 2–4 days	41.3(9.3)	44(35–45)	
Once per week	46.0(9.4)	45(38–55)	
Do you feel tired after each dialysis session?			0.001
Yes	52.2(8.7)	54(46–57)	
Sometimes/often	41.9(7.6)	40(36–47)	
When do you feel most tired?			0.016
Morning/noon	46.9(10.6)	46(37–57)	
Afternoon	41.8(8.1)	40(37–47)	
Night	47.6(8.6)	48(39–56)	
What is the duration of fatigue?			0.001
Continuous	56.2(7.0)	57(53–60)	
Some hours	43.5(8.2)	43(37–50)	
Do you feel pain during vein puncture?			0.108
No	43.5(10.0)	40(36–48)	
Rarely	43.0(7.3)	41(37–48)	
Sometimes	47.6(9.0)	48(40–56)	
Often/yes	46.4(10.6)	48(36–53)	
Do you think there is a change in body image after the diagnosis of the disease?			0.001
Yes	48.0(9.1)	48(40–56)	
No	38.0(4.8)	37(34–41)	
Have you had itching in your body since you started dialysis?			0.001
No	41.6(8.6)	39(36–46)	
Rarely/sometimes	46.4(8.7)	46(39–55)	
Often/yes	51.6(9.0)	53(43–58)	
Do you have muscle cramps?			0.606
No	43.9(8.6)	41(38–49)	
Rarely/sometimes	45.7(9.0)	45(38–55)	
Yes/often	46.8(11.8)	46(35–57)	
Do you have stiffness in your joints?			0.001
No	39.3(7.1)	38(35–44)	
Rarely/sometimes	45.6(8.9)	45(38–53)	
Yes/often	53.0(6.3)	56(47–58)	
Do you feel nauseous and vomiting?			0.003
No	42.8(8.7)	40(37–47)	
Rarely/sometimes	48.7(9.0)	50(43–56)	
Are you concerned about the fact that you now have a limited social life compared to what you had before you started dialysis?			0.637
Yes/sometimes	45.4(9.0)	45(38–53)	
No	45.0(11.4)	44(36–50)	
Are there any changes in your role as a husband/wife?			0.056
Yes/sometimes	44.2(8.5)	44(38–51)	
No	50.1(11.1)	49(36–58)	
Are you worried about the possibility of the dialysis machine shutting down?			0.939
Yes/sometimes	45.2(8.3)	45(38–52)	
No	45.5(10.3)	44(37–55)	
Do you have any restrictions on the clothes you can wear?			0.001
Yes/sometimes	47.5(8.8)	48(40–55)	
No	41.4(9.0)	39(35–45)	
Do you want to hide or conceal your body or any part of your body? (like the fistula)			0.151
Yes/sometimes	46.4(8.7)	45(39–53)	
No	44.1(9.8)	43(36–53)	
	Spearman’s Rho	p-value	
Recent urea	−0.081	0.423	
Recent creatinine	−0.050	0.619	
Recent phosphorus	0.158	0.115	
Physical fatigue	0.824	0.001	
Mental fatigue	0.746	0.001	
Insomnia (AIS)	0.707	0.001	

Effect of patients' characteristics on depression scale

Multiple linear regression was performed to evaluate the effect of patient characteristics on depression. Divorced/widowed participants had a statistically significantly higher depression score of 3.4 points than married patients (β = 3.42, 95% CI: 1.01-5.83, p = 0.006). In addition, patients who sometimes had tiredness after hemodialysis had a statistically significantly lower depression score of 2.8 points than those who had tiredness after each session (β = −2.77, 95% CI: −5.18 to −0.36, p = 0.025). Participants who did not believe that they had a change in body image had a 3.3 points lower depression score than those who believed that they had (β = −3.3, 95% CI: −5.62 to −0.98, p = 0.006). In addition, 1-point increase in the score of fatigue either physically or mentally indicated an increase of depression score by 0.35 and 0.31 points, respectively (β = 0.35, 95% CI: 0.10-0.65, p = 0.009 and β = 0.31, 95% CI: 0.08-0.55, p = 0.010, respectively). Likewise, a 1-point increase in the score of insomnia indicated an increase of depression score by 2.5 points (β = 2.53, 95% CI: 0.20-4.85, p = 0.034) (Table 3).

**Table 3:** Effect of patients' characteristics on the depression scale

	β coefficient (95% CI)	p-value
Age (years)		
≤60	Ref. Cat.	
61–70	1.43(−​​​​​​​1.37 to −​​​​​​​4.23)	0.314
>70	0.51(−​​​​​​​2.76 to −​​​​​​​3.79)	0.755
Status		
Married/living together	Ref. Cat.	
Single	1.33(−​​​​​​​1.69 to −​​​​​​​4.35)	0.384
Divorced/widowed	3.42(1.01–​​​​​​​5.83)	0.006
Job		
Unemployed/household	Ref. Cat.	
Employee	−2.94(−6.27 to −0.38)	0.082
Pensioner	−0.30(−3.24 to −2.63)	0.839
Other diseases		
Yes	Ref. Cat.	
No	−0.34(−2.56 to −1.87)	0.758
Do you believe that regular updating helps reduce stress?		
Very	Ref. Cat.	
Enough	−1.87(−4.02 to −0.27)	0.086
A little/not at all	−1.26(−3.98 to −1.45)	0.357
Do you feel tired after each dialysis session?		
Yes	Ref. Cat.	
Sometimes/often	−2.77(−5.18 to −0.36)	0.025
Do you think there is a change in body image after the diagnosis of the disease?		
Yes	Ref. Cat.	
No	−3.30(−5.62 to −0.98)	0.006
Physical fatigue	0.35(0.10–0.65)	0.009
Mental fatigue	0.31(0.08–0.55)	0.010
Insomnia (AIS)	2.53(0.20–4.85)	0.034

## Discussion

In the present study, it was found that participants over the age of 70 had a statistically significantly higher score of depression than younger participants. Similarly, in a previous study, it was found that old age was strongly related to increased levels of depression among patients on hemodialysis [11].

It can be assumed that elderly patients on hemodialysis usually suffer from chronic diseases such as diabetes, hypertension, and heart failure [4], leading to a high prevalence of physical symptoms and increased levels of depression [12]. Additionally, physical weakness can cause a lack of energy, mood disorders, and depression due to limited participation in social activities and social isolation [13]. Predialysis patients usually have significant muscle atrophy, which is the main factor for weakness [14]. Similarly, older patients on hemodialysis are gradually isolated from their social environment due to comorbid diseases, deaths of family members, physical weakness, and hearing loss [15].

Moreover, the findings of this study showed that high levels of depression were experienced by patients who did not wish to receive written information about the management of their disease. It can be assumed that a lack of information can lead to low levels of understanding of medical instructions [16]. Thus, patients with inadequate knowledge about their disease frequently miss dialysis sessions and do not comply with fluid and diet restrictions [17]. It is important to take into consideration that high levels of non-adherence can lead to malnutrition and increased levels of depression in dialysis patients [18].

Additionally, the findings of this study indicated that divorced/widowed patients had statistically significantly higher levels of depression. This can be attributed to the fact that patients undergoing hemodialysis often experience disorders in marital relationships since the chronic disease causes tension, negative emotions, and changes in marital roles [19]. In a similar study, it was found that older patients who were single, widowed, or divorced had high rates of depression, possibly due to the lack of support from their partners [20].

The present study also found that patients who had comorbid diseases had statistically significantly high levels of depression. It can be assumed that frequent hospitalizations are an important factor in depression in these patients [21] due to vascular disorders [22], hypertension, cardiovascular disease, musculoskeletal disorders, and diabetes mellitus [23]. Similarly, Lacson et al. found that depression in hemodialysis patients was significantly related to increased hospital admissions [24].

The present study also found that pensioners had statistically high levels of depression. This can be attributed to the fact that retirement usually leads to social isolation in older adults, which is a significant factor in depression [15]. Additionally, retirement has a close relationship with decreased working ability in these patients, since working-age individuals on hemodialysis have difficulties in maintaining employment due to co-morbid diseases, increased fatigue, frequent hospitalizations, and the long duration of hemodialysis sessions, each lasting four hours, three times a week [3]. Thus, a large number of them are early retired or reduce their working hours after the initiation of hemodialysis [25]. In a similar study, it was found that only a low percentage of dialysis patients (11.8%) had active employment due to retirement-related to their disease [26].

This study also showed that an increase in physical or mental fatigue and an increase in insomnia score indicated an increase in depression levels. Insomnia causes daytime sleepiness and lack of energy, which is regarded as a significant factor for fatigue [6]. It can be assumed that lack of energy leads to low physical well-being and social isolation, increasing depressive disorders in dialysis patients [13].

The results of this study offer significant information to health professionals about the factors related to depression in patients undergoing hemodialysis. Therefore, regular psychiatric evaluation of these patients and evaluation of risk factors are essential for the early diagnosis of depression and for reducing morbidity and mortality in these people.

Limitations of the study

The sample used in this study is a convenience sample since it comes from one renal unit in Athens, Greece. Thus, the findings cannot be generalized. Additionally, further investigation of the factors related to depression in hemodialysis patients can be achieved with more qualitative approaches.

## Conclusions

Depression is a common problem among hemodialysis patients and has a strong association with insomnia and fatigue. The findings of this study showed that depression had a strong association with a variety of demographic factors. Additionally, an increase in fatigue score and an increase in insomnia score indicated an increase in depression levels. Therefore, depression, insomnia, and fatigue should be early assessed and effectively treated by renal professionals in order to improve the quality of life of these people and reduce their morbidity and mortality.
